# Chiroptical Symmetry Analysis: Exciton Chirality-Based Formulae to Understand the Chiroptical Responses of *C*_n_ and *D*_n_ Symmetric Systems

**DOI:** 10.3390/molecules24010141

**Published:** 2019-01-01

**Authors:** Silvia Castro-Fernández, Ángeles Peña-Gallego, Ricardo A. Mosquera, José Lorenzo Alonso-Gómez

**Affiliations:** 1 Departamento de Química Orgánica, Facultade de Químicas, Universidade de Vigo, Lagoas-Marcosende s/n, 36310 Vigo, Spain; castrofsilvia@gmail.com; 2 Departamento de Química Física, Facultade de Químicas, Universidade de Vigo, Lagoas-Marcosende s/n, 36310 Vigo, Spain; mpena@uvigo.es

**Keywords:** chirality, chiroptical responses, exciton chirality, selection rules, symmetry

## Abstract

The high sensitivity of chiroptical responses to conformational changes and supramolecular interactions has prompted an increasing interest in the development of chiroptical applications. However, prediction of and understanding the chiroptical responses of the necessary large systems may not be affordable for calculations at high levels of theory. In order to facilitate the development of chiroptical applications, methodologies capable of evaluating the chiroptical responses of large systems are necessary. The exciton chirality method has been extensively used for the interaction between two independent chromophores through the Davydov model. For systems presenting *C*_2_ or *D*_2_ symmetry, one can get the same results by applying the selection rules. In the present article, the analysis of the selection rules for systems with symmetries *C*_n_ and *D*_n_ with n = 3 and 4 is used to uncover the origin of their chiroptical responses. We foresee that the use of the Chiroptical Symmetry Analysis (CSA) for systems presenting the symmetries explored herein, as well as for systems presenting higher symmetries will serve as a useful tool for the development of chiroptical applications.

## 1. Introduction

Chirality, or the existence of a pair of non-superimposable mirror-image shapes, is a natural property present in a wide variety of biological systems. While enantiomeric molecules present the same interaction with other achiral molecules, they may interact differently with other chiral molecules or entities. Besides its impact in the biological world being ubiquitous [1,2], chirality can also be exploited in other areas such as materials science. For example, the interaction of chiral compounds with light has been used for the construction of light-powered molecular motors [3] or in the development of more sensitive and specific sensors [4,5]. The increasing interest in the potentiality of chiral systems for miscellaneous applications makes essential the understanding of their chiroptical responses and the development of methodologies to study them.

When linearly-polarized light passes through a chiral medium, the resulting light can become elliptically polarized due to the different absorption of the right and left circularly-polarized light by the chiral medium [6]. This phenomenon is known as circular dichroism, which can exist in two forms, electronic (UV-visible range) or vibrational (IR range). Derived from the particular interaction of chiral molecules with light, optical methodologies were developed to characterize enantiomers according to their absolute configurations [7].

**Scheme 1:**
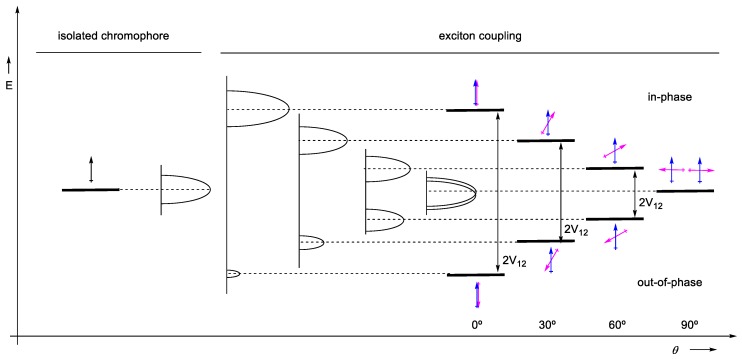
Representation of the splitting of the energy excitation of two interacting chromophores due to the in-phase and out-of-phase interactions. Arrows represent the electronic transition for two chromophores, blue and magenta. Curves represent the absorption band in the UV-Vis spectra. Different torsion angles between the two chromophores lead to different relative intensities and Δ*E* between the two possible transitions.

Electronic Circular Dichroism (ECD) is one of the most used chiroptical spectroscopies [8,9]. Comparison of the experimental and theoretically-predicted ECD spectra is routinely used not only for absolute and relative configuration assignment of small and medium-sized molecules [10], but also for elucidating the conformational preferences of the systems under study [11,12]. The level of theory employed for the simulation of the ECD spectra must be carefully chosen according to the nature of the systems under study [13]. As an alternative to the use of ab initio calculations, the exciton chirality method can be applied when two independent chromophores are present in the molecule [14]. The more general phenomenon known as the Davydov splitting [15], 2*V*_12_, emerges from the excitation energy splitting between the in-phase and out-of-phase simultaneous two-chromophore transitions, strongly dependent on their relative orientation (Scheme 1).

**Scheme 2:**
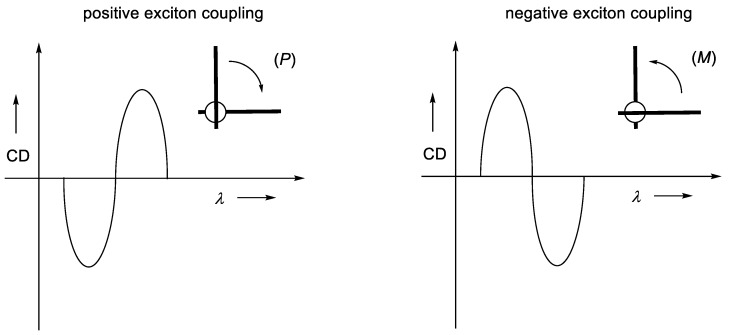
Representation of the bisignated CD signal arising from the interaction between two independent chromophores with a relative positive (**left**) or negative (**right**) twist.

When the arrangement between the interacting chromophores is chiral, the Cotton effects emerging from the in-phase and out-of-phase excitations in the CD spectrum present opposite signs. Notable, the sign of the less energetic band in the resulting bisignated CD signal is diagnostic of the handedness of the chiral arrangement between the chromophores, allowing the absolute configuration determination of many compounds [14,16] (Scheme 2).

The Davydov model considers an ideal system formed by two non-conjugated chromophores with two-fold degenerate excited states. Perturbation theory allows breaking degeneracy considering the interaction between two chromophores. If two chromophores are represented by the corresponding Electric Dipole Transition Moment (EDTM), ${\vec{\mu }}_{i}^{t}$, the interaction between them, *V*_12_, is given (in au) by Equation (1), where ${\vec{R}}_{12}$ is the vector connecting the origin of ${\vec{\mu }}_{1}^{t}$ and ${\vec{\mu }}_{2}^{t}$. When these chromophores display also permanent dipoles in the excited or ground state, represented respectively by ${\vec{\mu }}_{i}^{0}$ and ${\vec{\mu }}_{i}^{a}$ additional terms have to be considered within *V*_12_, as will be shown below.

(1) $$V_{12}=R_{12}{}^{-3}\left[{\vec{\mu }}_{1}^{t}\cdot {\vec{\mu }}_{2}^{t}-3R_{12}{}^{-2}\left({\vec{\mu }}_{1}^{t}\cdot {\vec{R}}_{12}\right)\left({\vec{\mu }}_{2}^{t}\cdot {\vec{R}}_{12}\right)\right]$$

The application of the perturbation theory to the (originally degenerate) first electronically-excited level yields an energy splitting, Δ*E*, between these two states (usually named α and β) given by Equation (2). In this article, we restrict this study to electrostatic interactions. We are aware of the importance of van der Waals interactions; however, it is not in the scope of this first approximation.

(2) $$\Delta E=2V_{12}$$

This outcome is comparable with the analysis of *C*_2_ and *D*_2_ symmetric systems in terms of the selection rules. In recent years, we have contributed with a number of highly symmetric systems such as linear [12] and cyclic oligomers [17], molecular containers [18,19], or even all-carbon double helices [20], presenting remarkable chiroptical responses in the search for valuable materials for everyday chiroptical applications [21]. However, analysis of the chiroptical responses is often tedious [22,23]. In this regard, in 1937, Condon [24] revised the work from Kirkwood [25], where he demonstrated the possibility to determine the optical activity of a molecule by considering the relative orientation of the different constituent groups. In 1974, Schellman developed the theory of optical activity with simple electric and magnetic dipole interaction with radiation capable of resampling rather accurately the experimental results [26]. In order to have a more accessible understanding of the behavior of highly symmetric systems, herein we perform the Chiroptical Symmetry Analysis (CSA) and apply it to systems with *C*_n_ and *D*_n_ symmetries for n = 2, 3, and 4. Even when this approach is more specific, CSA could be applied to any other symmetry, and therefore, we hope that it will serve as a design tool for the development of everyday chiroptical applications in the near future. Additionally, since an exciton coupling approach in Vibrational CD (VCD), known as the coupled oscillator model, has also been employed successfully by Monde [27] and further evaluated by Polavarapu [28], in analogy to electronic transition, the CSA could also be applied for the prediction of the VCD of highly symmetric systems.

## 2. Results and Discussion

### 2.1. Theoretical Background (Binary Systems)

**Scheme 3:**
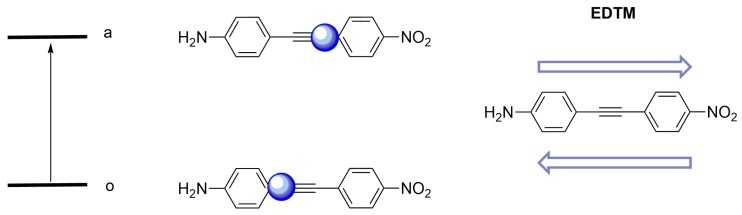
Representation of the *o*→*a* electronic excitation of a model compound. The electron density redistribution from the ground state *o* to the excited state *a* results in the Electric Dipole Transition Moment (EDTM). EDTM has a defined direction and two equally-probable orientations. The blue spheres represent the center of electron density for the electronic states.

The interaction of UV-visible light with a molecule can result in an electric excitation, usually depicted, within the Hartree–Fock framework, as the transition from one of the occupied molecular orbitals, *Φ*_i_^0^, to an empty (virtual) one, *Φ*_j_^a^. This produces a rearrangement of the electron density of the molecule, basically described by the EDTM as a linear displacement of electron density during the transition (Scheme 3). Moreover, the intensity of the transition between the two states is proportional to the square of the modulus of the EDTM, and forbidden electronic transitions yield null values of EDTM. The selection rules establish that for a transition to be allowed, at least one of the components of the EDTM must contain the totally-symmetric representation of the system point group, and consequently, the direct product for the representations of the involved wave functions must contain at least one of the representations of the electric dipole moment components.

Davydov’s molecular exciton model provides an elegant explanation for the CD Cotton effects in a molecule with two identical chromophores [14]. The model predicts that dipole interaction between chromophores splits the ideally degenerate excited states. Neglecting all kinds of interactions between the chromophores (zero-order approximation), the relative energy of the excited states with regard to the ground state would be the same as in an isolated chromophore, *E*^a^. In fact, when considering that the electronic transition only affects the chromophores, it is assumed that the rest of the molecule remains unchanged by the transition. We consider it to be worth reviewing this model with a certain level of detail before expanding it to three and four chromophores.

#### 2.1.1. Approximated Wave Functions and Energy Levels 

According to Davydov’s model, in the zero-order approach, the ground state, *Ψ*^0(0)^, can be represented by the product of two ground state wave functions for the isolated chromophore (3), *Ψ*^0^. In the same vein, the zero-order degenerate excited electronic state, *Ψ*^a(0)^, can be expressed by the product of ground and excited states, *Ψ*^a^, of single chromophores, with two equivalent possibilities: *Ψ*_1_^a(0)^ (4) and *Ψ*_2_^a(0)^ (5). The former refers to the chromophore 1 in the ground state and the chromophore 2 in the excited state. This assumes that the electronic excitation only modifies the electron density distribution of the chromophore where it takes place.

(3) $$\Psi^{0\left(0\right)}=\psi^{0}\psi^{0}$$

(4) $$\Psi_{1}^{a\left(0\right)}=\psi^{0}\psi^{a}$$

(5) $$\Psi_{2}^{a\left(0\right)}=\psi^{a}\psi^{0}$$

The application of the perturbation theory to the ground state (non-degenerated) provides for the first-order correction to the energy, *E*^0(1)^, the usual expression given by Equation (6). Considering only the dipole-dipole interaction between the chromophores, the first-order correction for the energy of the ground state is that shown by Equation (7), where ${\vec{\mu }}_{i}^{0}$ are the dipole moments of the chromophores in the ground state, named $V_{12}^{00}$.

(6) $$E^{0\left(1\right)}=\langle \psi^{0}\psi^{0}\left|{\hat{V}}_{12}\right|\psi^{0}\psi^{0}\rangle$$

(7) $$E^{0\left(1\right)}=V_{12}^{00}=R_{12}{}^{-3}\left[{\vec{\mu }}_{1}^{0}\cdot {\vec{\mu }}_{2}^{0}-3R_{12}{}^{-2}\left({\vec{\mu }}_{1}^{0}\cdot {\vec{R}}_{12}\right)\left({\vec{\mu }}_{2}^{0}\cdot {\vec{R}}_{12}\right)\right]$$

Obtaining the first-order correction for the energy of the degenerated states leads to a set of linear homogenous equations, with a non-trivial solution only when the condition (8) is verified. *E*^a(1)^ is the first-order correction to the energy of the excited state for the two-chromophore system. Condition (8) is only fulfilled by the two *E*^a(1)^ values contained in Expression (9). We observe that these values involve the dipole–dipole interaction between one chromophore in the ground state and another in the excited state, $V_{12}^{0a}$, and a term where the interaction applies over the electronic transition, $V_{12}^{tt}$, shown in (10).

(8) $$\left|\begin{array}{cc} \langle \psi^{0}\psi^{a}\left|{\hat{V}}_{12}\right|\psi^{0}\psi^{a}\rangle -E^{a\left(1\right)} & \langle \psi^{0}\psi^{a}\left|{\hat{V}}_{12}\right|\psi^{a}\psi^{0}\rangle \\ \langle \psi^{a}\psi^{0}\left|{\hat{V}}_{12}\right|\psi^{0}\psi^{a}\rangle & \langle \psi^{a}\psi^{0}\left|{\hat{V}}_{12}\right|\psi^{a}\psi^{0}\rangle -E^{a\left(1\right)} \end{array}\right|=0$$

(9) $$E^{a\left(1\right)}=V_{12}^{0a}\pm V_{12}^{tt}$$

(10) $$V_{12}^{tt}=\langle \psi^{0}\psi^{a}\left|{\hat{V}}_{12}\right|\psi^{a}\psi^{0}\rangle =R_{12}{}^{-3}\left[{\vec{\mu }}_{1}^{t}\cdot {\vec{\mu }}_{2}^{t}-3R_{12}{}^{-2}\left({\vec{\mu }}_{1}^{t}\cdot {\vec{R}}_{12}\right)\left({\vec{\mu }}_{2}^{t}\cdot {\vec{R}}_{12}\right)\right]$$

Thus, after introducing the first-order corrections obtained with perturbation theory for ground and excited electronic states, the relative energy of the excited state, Δ*E*^a^, can be expressed by Equation (11). Obviously, the reliability of the formula can be improved including further corrections, but the most significant fact is the splitting of the excited level into two different states (usually named α and β), whose energy differs in $2V_{12}^{tt}$. Moreover, replacing the two solutions for energy correction (9) in the system of homogeneous equations and normalizing, it is found that the zero-order wave functions for α and β states follow, respectively, Expressions (12) and (13).

(11) $$\Delta E^{a}≅\Delta E^{a\left(0\right)}+\Delta E^{a\left(1\right)}=E^{a}+V_{12}^{0a}-V_{12}^{00}\pm V_{12}^{tt}$$

(12) $$\psi_{\alpha }^{\left(0\right)}=\frac{1}{\sqrt{2}}\left(\psi^{a}\psi^{0}-\psi^{0}\psi^{a}\right)$$

(13) $$\psi_{\beta }^{\left(0\right)}=\frac{1}{\sqrt{2}}\left(\psi^{a}\psi^{0}+\psi^{0}\psi^{a}\right)$$

**Scheme 4:**
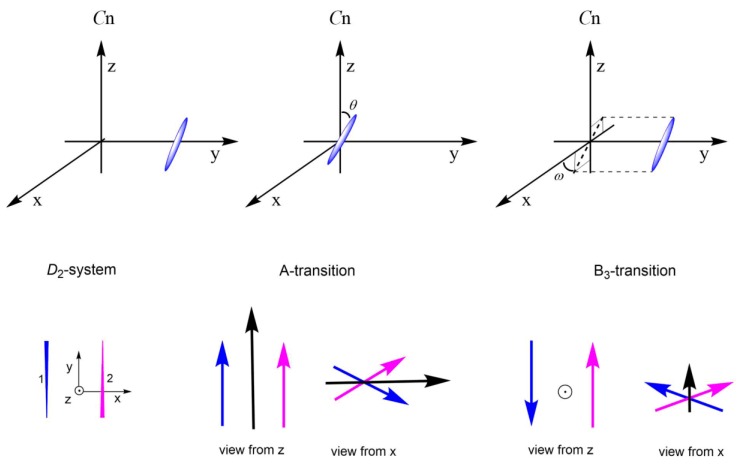
**Top**: Representation of angles to define the position of the chromophores. **Bottom**: Representation of a system presenting *D*_2_ symmetry. According to the selection rules, the two equivalent chromophores 1 (blue) and 2 (magenta) can undergo a simultaneous excitation following A or B_3_ symmetry leading to total EDTMs (black) parallel to y or z, respectively, as a result of the summation of the EDTM of each chromophore. A circle with a dot in the middle represents an arrow perpendicular to the plane pointing to the reader.

One can directly obtain wave functions (12) and (13) by means of group theory if chromophores are in the *C*_2_ arrangement. In this case, energies for α and β states (named, respectively, A and B according to symmetry) can be calculated as average values (Scheme 4) (14).

(14) $$\langle \psi_{\beta }^{\left(0\right)}\left|{\hat{V}}_{12}\right|\psi_{\beta }^{\left(0\right)}\rangle =\langle \psi_{\alpha }^{\left(0\right)}\left|{\hat{V}}_{12}\right|\psi_{\alpha }^{\left(0\right)}\rangle +2V_{12}^{tt}$$

#### 2.1.2. Rotatory Strength

According to Rosenfeld´s quantum theory [29], the rotatory strength, *R*, defined as the difference between the absorption rate of Left Circularly-Polarized light (LCP) and Right Circularly-Polarized light (RCP) in a certain transition, is given by (15), where ${\hat{\mu }}_{e}$ and ${\hat{\mu }}_{m}$ are, respectively, the operators for electric (16) and magnetic dipole moment (17) in a system formed by *J* chromophores, each of them with *K* electrons. *r_jk_* and *p_jk_* are, respectively, the position and linear momentum vectors for electron *k* in chromophore *j*. Both are the summation of one-electron operators. Within the context of the independent exciton model, the summation only involves the *N* electrons provided by the chromophores, and these electrons can be assigned to a certain chromophore *j*.

(15) R=Im[〈Ψ0|μ^e|Ψa〉⋅〈Ψa|μ^m|Ψ0〉]

(16) $${\hat{\mu }}_{e}=e\sum_{j=1}^{J}\sum_{k=1}^{K}{\vec{r}}_{jk}$$

(17) $${\hat{\mu }}_{m}=\frac{e\hbar }{2mci}\sum_{j=1}^{J}\sum_{k=1}^{K}{\vec{r}}_{jk}\times \nabla_{jk}=\frac{e}{2mc}\sum_{j=1}^{J}\sum_{k=1}^{K}{\vec{r}}_{jk}\times {\hat{\vec{p}}}_{jk}$$

Rosenfeld’s equation states that *R* depends on the scalar product of the EDTM and the corresponding Magnetic Transition Dipole Moment (MDTM), both derived below. Consequently, optical activity in a certain transition implies: (i) transition is both electric dipole and magnetic dipole allowed; and (ii) EDTM and MDTM for the process are not mutually orthogonal.

#### 2.1.3. Electric Dipole Transition Moment

The fact that EDTMs are origin independent and in the form of Equation (16) allows defining an EDTM vector for each chromophore, ${\vec{\mu }}_{j}^{t}$, (18). The molecular EDTM results in a specific combination of individual vectors for each particular electronic transition. In the case of a two-chromophore system, the first electronically-excited states, labeled as α and β, are represented in the zero-order approach, respectively, by wave functions (12) and (13) and the ground state by (3). Application of simple algebra (for more information, see the Supporting Information) leads to molecular EDTM, which follows Expression (19) in the α state and Expression (20) in the β state. The square modulus of the molecular EDTM (directly related to Einstein coefficients in EDTM transitions) depends, in both cases, on the angle between these vectors, *γ*. Thus, in the case of two identical chromophores, Expressions (21) and (22) are respectively followed for transitions to α and β states. Let us now think about the *γ* angle. For two isolated identical chromophores, the three components of their vectors will be equal. The only difference in the independent exciton model is that the chromophores are placed in specific positions in the molecule, introducing different orientation for the groups. The *γ* angle is the one measuring this relative orientation. As a consequence, when the two chromophores are not orthogonally arranged in the molecule, the absorptions for 0→α and 0→β transitions are of different intensity.

(18) $${\vec{\mu }}_{j}^{t}=e\sum_{k}\langle \psi^{0}\left({\vec{r}}_{jk}\right)\left|{\vec{r}}_{jk}\right|\psi^{a}\left({\vec{r}}_{jk}\right)\rangle$$

(19) $${\vec{\mu }}^{t\alpha }=\frac{{\vec{\mu }}_{1}^{t}-{\vec{\mu }}_{2}^{t}}{\sqrt{2}}$$

(20) $${\vec{\mu }}^{t\beta }=\frac{{\vec{\mu }}_{1}^{t}+{\vec{\mu }}_{2}^{t}}{\sqrt{2}}$$

(21) $${\left|{\vec{\mu }}^{t\alpha }\right|}^{2}={\left|{\vec{\mu }}_{1}^{t}\right|}^{2}\left(1-\cos\gamma \right)$$

(22) $${\left|{\vec{\mu }}^{t\beta }\right|}^{2}={\left|{\vec{\mu }}_{1}^{t}\right|}^{2}\left(1+\cos\gamma \right)$$

#### 2.1.4. Magnetic Dipole Transition Moment

Within the hypothesis of the independent exciton model, the molecular MDTM for a certain transition (*t*: 0→*a*) contained in Rosenfeld’s expression can be obtained as the vector summation of the MDTMs corresponding to each chromophore, *j*. The MDTM of each chromophore is represented by a vector, ${\vec{M}}_{j}^{t}$, defined by Expression (23), where subscript *k* refers to the electrons in chromophore *j*.

(23) $${\vec{M}}_{j}^{t}=\frac{e}{2mc}\sum_{k}\langle \psi^{0}\left({\vec{r}}_{jk}\right)\left|{\vec{r}}_{jk}\times {\hat{\vec{p}}}_{jk}\right|\psi^{a}\left({\vec{r}}_{jk}\right)\rangle$$

${\vec{M}}_{j}^{t}$ vectors are origin dependent. Thus, their summation should be done considering a common origin for all the electron position vectors, ${\vec{r}}_{jk}$. Introducing a reference point for each chromophore (e.g., its center of mass), with position vector ${\vec{R}}_{j}$, and the relative positions for the electrons in their chromophore, ${\vec{r}}_{jk}^{\prime }$, (23) turns into (24), where ${\vec{M}}_{j}^{t}$ splits into two parts.

(24) $${\vec{M}}_{j}^{t}=\frac{e}{2mc}\left[{\vec{R}}_{j}\times \sum_{k}\langle \psi^{0}\left({\vec{r}}_{jk}\right)\left|{\hat{\vec{p}}}_{jk}\right|\psi^{a}\left({\vec{r}}_{jk}\right)\rangle \right]+\frac{e}{2mc}\sum_{k}\langle \psi^{0}\left({\vec{r}}_{jk}\right)\left|{\vec{r}}_{jk}^{\prime }\times {\hat{\vec{p}}}_{jk}\right|\psi^{a}\left({\vec{r}}_{jk}\right)\rangle$$

The first addend contains the linear transition moment of the chromophore, ${\vec{p}}_{j}^{t}$, defined by (25), whereas the second one (internal MDTM) defines the MDTM of the isolated chromophore, ${\vec{m}}_{j}^{t}$, (26). This allows one to relate (Equation (27)) the MDTM of the chromophore in the molecule, ${\vec{M}}_{j}^{t}$, with its value in an isolated species, ${\vec{m}}_{j}^{t}$.

(25) $${\vec{p}}_{j}^{t}=\sum_{k}\langle \psi^{0}\left({\vec{r}}_{jk}\right)\left|{\hat{\vec{p}}}_{jk}\right|\psi^{a}\left({\vec{r}}_{jk}\right)\rangle$$

(26) $${\vec{m}}_{j}^{t}=\frac{e}{2mc}\sum_{k}\langle \psi^{0}\left({\vec{r}}_{jk}\right)\left|{\vec{r}}_{jk}^{\prime }\times {\hat{\vec{p}}}_{jk}\right|\psi^{a}\left({\vec{r}}_{jk}\right)\rangle$$

(27) $${\vec{M}}_{j}^{t}=\frac{e\left({\vec{R}}_{j}\times {\vec{p}}_{j}^{t}\right)}{2mc}+{\vec{m}}_{j}^{t}$$

The replacement of ${\vec{p}}_{j}^{t}$ in terms of the EDTM of the isolated chromophore,${\vec{\mu }}_{j}^{t}$ (for more information, see the Supporting Information), leads to Equation (28), where ${\bar{\nu }}^{t}$ is the wave number for the transition.

(28) $${\vec{M}}_{j}^{t}=i\pi {\bar{\nu }}^{t}\left({\vec{R}}_{j}\times {\vec{\mu }}_{j}^{t}\right)+{\vec{m}}_{j}^{t}$$

At this point, the summation of ${\vec{M}}_{j}^{t}$ vectors leads to molecular MDTM. This is shown for a binary system in states α and β in Equations (29) and (30).

(29) $${\vec{M}}^{t\alpha }=\frac{1}{\sqrt{2}}\left[i\pi {\bar{\nu }}^{t}\left({\vec{R}}_{1}\times {\vec{\mu }}_{1}^{t}-{\vec{R}}_{2}\times {\vec{\mu }}_{2}^{t}\right)+\left({\vec{m}}_{1}^{t}-{\vec{m}}_{2}^{t}\right)\right]$$

(30) $${\vec{M}}^{t\beta }=\frac{1}{\sqrt{2}}\left[i\pi {\bar{\nu }}^{t}\left({\vec{R}}_{1}\times {\vec{\mu }}_{1}^{t}+{\vec{R}}_{2}\times {\vec{\mu }}_{2}^{t}\right)+\left({\vec{m}}_{1}^{t}+{\vec{m}}_{2}^{t}\right)\right]$$

#### 2.1.5. Expressions for Rotatory Strength

Introducing EDTM and MDTM expressions obtained for α ((19) and (29)) and β ((20) and (30)) states of a binary system within Rosenfeld’s expression (15) leads to obtaining the rotatory strength for both transitions, (31) and (32), respectively.

(31) Rα=12Im[(μ→1−μ→2)⋅(m→1−m→2)]−πν¯t2(R→2−R→1)(μ→1×μ→2)

(32) Rβ=12Im[(μ→1+μ→2)⋅(m→1+m→2)]+πν¯t2(R→2−R→1)(μ→1×μ→2)

In what follows, we restrict ourselves to those cases (e.g., π→π* transitions in simple chromophores such as ethene or benzene) where internal MDTM are negligible, and therefore, the first addend can be disregarded from Equations (31) and (32). In those cases, the Cotton effects for transitions to α and β states display the same intensity with opposite signs ((33) and (34)).

(33) $$R^{\alpha }=-\frac{\pi {\bar{\nu }}^{t}}{2}\left({\vec{R}}_{2}-{\vec{R}}_{1}\right)\left({\vec{\mu }}_{1}\times {\vec{\mu }}_{2}\right)$$

(34) $$R^{\beta }=\frac{\pi {\bar{\nu }}^{t}}{2}\left({\vec{R}}_{2}-{\vec{R}}_{1}\right)\left({\vec{\mu }}_{1}\times {\vec{\mu }}_{2}\right)$$

These expressions are usually present in chiroptical spectroscopy reviews and monographies [7]. Taking into account symmetry, e.g., *C*_2_, a further step can be done, and the values of rotatory strength can be predicted in terms of simple geometry parameters. Thus, for *C*_2_ symmetry, a convenient reference system for the vectorial operations indicated above has its origin at the center of mass; the z axis is given by the *C*_2_ axis; and x axis connects the molecular center of mass with that of Chromophore 1. We notice that the orientation of the EDTMs is given by two angles, which are formed with the *C*_2_ axis, coincident with the z axis, *θ*, and the angle between the projection of EDTM for each chromophore on the zx plane with the x axis, ω. Thus, simple calculations lead to Expression (35), where rotatory strength is a function of the distance between chromophores, *R*_12_ or R as the distance from the center of the chromophores to the *C_2_*

(35) $$R^{\alpha }=\pi {\bar{\nu }}^{t}R_{12}{\left|{\vec{\mu }}_{1}\right|}^{2}\sin\left(\theta \right)\cos\left(\theta \right)\sin\left(\omega \right)=2\pi {\bar{\nu }}^{t}R{\left|{\vec{\mu }}_{1}\right|}^{2}\sin\left(\theta \right)\cos\left(\theta \right)\sin\left(\omega \right)=-R^{\beta }$$

### 2.2. Extending the Exciton-Independent Model to Systems with Three and Four Chromophores

In the following, we apply the independent exciton model to systems formed by three identical chromophores with point groups *C*_3_ (the system presents one *C*_3_ axis) and *D*_3_ (the system presents three *C*_2_ axes perpendicular to a *C*_3_ axis). Our aims are: (i) obtaining expressions for the splitting between those first electronically-excited states attainable from the ground state by absorption of electromagnetic radiation allowed by electric dipole transition; and (ii) predicting the corresponding rotatory strengths. 

#### 2.2.1. *C*_3_ Geometries

**Scheme 5:**
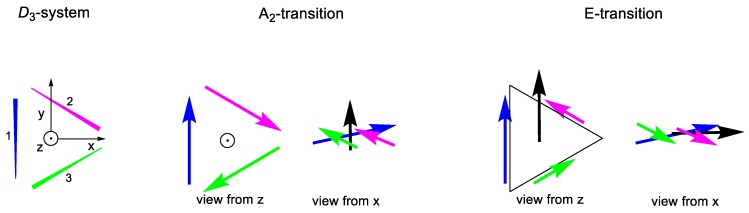
Representation of a system presenting *D*_3_ symmetry. According to the selection rules, the three equivalent chromophores 1 (blue), 2 (magenta), and 3 (green) can undergo a simultaneous excitation following A_2_ or E symmetry, leading to total EDTMs (black) parallel to z or y, respectively, as a result of the summation of the EDTM of each chromophore. A circle with a dot in the middle represents an arrow perpendicular to the plane pointing to the reader.

Assuming that the electronic structure of the three chromophores is unchanged with regard to the isolated one, the ground state wave functions could be zero-order approached by Equation (36) and belong to the totally-symmetric irreducible representation of this point group, A. We can expect three equivalent monoexcitations from the ground state, described by the wave functions shown in (37) (Scheme 5). They can be used as a basis set for constructing *C*_3_ Symmetry-Adapted Linear Combinations (SALC) for the first electronically-excited states. In this case, the reducible representation decomposes into the symmetry irreducible species A and the pseudodegenerate reducible species E, as shown in (38). Thus, one A excited state and two E excited electronic states can be predicted. They are represented in the zero-order approach, respectively, by Equations (39)–(41). Transitions A and E (E1 and E2) are both orbitally allowed.

(36) $$\Psi^{0\left(0\right)}=\psi^{0}\psi^{0}\psi^{0}$$

(37) $$\Psi_{1}^{a\left(0\right)}=\psi^{a}\psi^{0}\psi^{0},\;\Psi_{2}^{a\left(0\right)}=\psi^{0}\psi^{a}\psi^{0},\;\Psi_{3}^{a\left(0\right)}=\psi^{0}\psi^{0}\psi^{a}$$

(38) $$\Gamma \left(\Psi_{1}^{a\left(0\right)},\Psi_{2}^{a\left(0\right)},\Psi_{3}^{a\left(0\right)}\right)=A⊕E$$

(39) $$\Psi_{A}^{a\left(0\right)}=\frac{1}{\sqrt{3}}\left[\psi^{a}\psi^{0}\psi^{0}+\psi^{0}\psi^{a}\psi^{0}+\psi^{0}\psi^{0}\psi^{a}\right]$$

(40) $$\Psi_{E1}^{a\left(0\right)}=\frac{1}{\sqrt{3}}\left[\psi^{a}\psi^{0}\psi^{0}+\psi^{0}\psi^{a}\psi^{0}e^{2\pi i/3}+\psi^{0}\psi^{0}\psi^{a}e^{-2\pi i/3}\right],$$

(41) $$\Psi_{E2}^{a\left(0\right)}=\frac{1}{\sqrt{3}}\left[\psi^{a}\psi^{0}\psi^{0}+\psi^{0}\psi^{a}\psi^{0}e^{-2\pi i/3}+\psi^{0}\psi^{0}\psi^{a}e^{2\pi i/3}\right],$$

Considering that the interaction between the three chromophores is pair additive (42) and the interactions between all pairs are equal, the first-order corrections for the energies of the ground, A, and E states are respectively shown by (43)–(45). We highlight that, within the first-order approach, both pseudodegenerate states display the same energy (45). Thus, in this case, the excited level splitting is $3V_{12}^{tt}$ and therefore can be also calculated by means of Davydov´s equation.

(42) $$\hat{V}={\hat{V}}_{12}+{\hat{V}}_{13}+{\hat{V}}_{23}$$

(43) $$E^{0\left(1\right)}=3V_{12}^{00}$$

(44) $$E_{A}^{a\left(1\right)}=2V_{12}^{a0}+V_{12}^{00}+2V_{12}^{tt}$$

(45) $$E_{E}^{a\left(1\right)}=2V_{12}^{a0}+V_{12}^{00}-V_{12}^{tt}$$

The corresponding EDTMs for transitions A, (46), and E, (47) and (48), are straightforwardly obtained extending to three excitons the procedure detailed in Section 2.1.3.

(46) $${\vec{\mu }}^{tA}=\frac{{\vec{\mu }}_{1}^{t}+{\vec{\mu }}_{2}^{t}+{\vec{\mu }}_{3}^{t}}{\sqrt{3}}$$

(47) $${\vec{\mu }}^{tE1}=\frac{1}{\sqrt{3}}\left({\vec{\mu }}_{1}^{t}+e^{2\pi i/3}{\vec{\mu }}_{2}^{t}+e^{-2\pi i/3}{\vec{\mu }}_{3}^{t}\right)=\frac{1}{\sqrt{3}}\left({\vec{\mu }}_{1}^{t}-\frac{{\vec{\mu }}_{2}^{t}+{\vec{\mu }}_{3}^{t}}{2}\right)+\frac{i}{2}\left({\vec{\mu }}_{2}^{t}-{\vec{\mu }}_{3}^{t}\right)$$

(48) $${\vec{\mu }}^{tE2}=\frac{1}{\sqrt{3}}\left({\vec{\mu }}_{1}^{t}+e^{-2\pi i/3}{\vec{\mu }}_{2}^{t}+e^{2\pi i/3}{\vec{\mu }}_{3}^{t}\right)=\frac{1}{\sqrt{3}}\left({\vec{\mu }}_{1}^{t}-\frac{{\vec{\mu }}_{2}^{t}+{\vec{\mu }}_{3}^{t}}{2}\right)-\frac{i}{2}\left({\vec{\mu }}_{2}^{t}-{\vec{\mu }}_{3}^{t}\right)$$

A convenient reference system for performing vectorial combinations of the EDTMs is that where the origin is at the center of mass, the z axis is given by the *C*_3_ axis, and the x axis connects the molecular center of mass with that of Chromophore 1. We notice that the orientation of the EDTMs is given by two angles, which are formed with the *C*_3_ axis, *θ*, and the angle between the projection of EDTM for each chromophore on the xy plane with the x axis, ω for Chromophore 1. Thus, the total EDTM for the transition to the A state displays only one non-zero component. It corresponds to the symmetry axis (z axis by convention) as Equation (49) shows. Total EDTMs to E1 and E2 states are orthogonal to that of the A state and contain imaginary parts, as indicated in Expressions (50) and (51).

(49) $${\vec{\mu }}^{tA}=\sqrt{3}\left|{\vec{\mu }}_{1}^{t}\right|\cos\left(\theta \right)\vec{k}$$

(50) $${\vec{\mu }}^{tE1}=\frac{\left|{\vec{\mu }}_{1}^{t}\right|\sin\theta }{2\sqrt{3}}\left[\left(\cos\omega -i3\sin\omega \right)\;\vec{i}+\left(\sin\omega +i3\cos\omega \right)\;\vec{j}\right]$$

(51) $${\vec{\mu }}^{tE2}=\frac{\left|{\vec{\mu }}_{1}^{t}\right|\sin\theta }{2\sqrt{3}}\left[\left(\cos\omega +i3\sin\omega \right)\;\vec{i}+\left(\sin\omega -i3\cos\omega \right)\;\vec{j}\right]$$

The corresponding MDTMs, (52) to (54), are obtained extending the procedure detailed in Section 2.1.4. and considering isolated chromophores with negligible internal MTDM.

(52) $${\vec{M}}^{tA}=\frac{i\pi {\bar{\nu }}^{t}}{\sqrt{3}}\left({\vec{R}}_{1}\times {\vec{\mu }}_{1}^{t}+{\vec{R}}_{2}\times {\vec{\mu }}_{2}^{t}+{\vec{R}}_{3}\times {\vec{\mu }}_{3}^{t}\right)$$

(53) $${\vec{M}}^{tE1}=\frac{i\pi {\bar{\nu }}^{t}}{\sqrt{3}}\left({\vec{R}}_{1}\times {\vec{\mu }}_{1}^{t}+e^{2\pi i/3}{\vec{R}}_{2}\times {\vec{\mu }}_{2}^{t}+e^{-2\pi i/3}{\vec{R}}_{3}\times {\vec{\mu }}_{3}^{t}\right)$$

(54) $${\vec{M}}^{tE2}=\frac{i\pi {\bar{\nu }}^{t}}{\sqrt{3}}\left({\vec{R}}_{1}\times {\vec{\mu }}_{1}^{t}+e^{-2\pi i/3}{\vec{R}}_{2}\times {\vec{\mu }}_{2}^{t}+e^{2\pi i/3}{\vec{R}}_{3}\times {\vec{\mu }}_{3}^{t}\right)$$

As a consequence, the rotatory strength for the transition to the A state is represented by Equation (55).

(55) $$R^{A}=\frac{-\pi {\bar{\nu }}^{t}}{3}\left[\left({\vec{R}}_{2}-{\vec{R}}_{1}\right)\left({\vec{\mu }}_{1}\times {\vec{\mu }}_{2}\right)+\left({\vec{R}}_{3}-{\vec{R}}_{1}\right)\left({\vec{\mu }}_{1}\times {\vec{\mu }}_{3}\right)+\left({\vec{R}}_{3}-{\vec{R}}_{2}\right)\left({\vec{\mu }}_{2}\times {\vec{\mu }}_{3}\right)\right]$$

which, taking into account the relationships imposed by *C*_3_ symmetry, results in (56), where R is the distance from the center of the chromophores to the C_3_ axis,

(56) $$R^{A}=-3\pi {\bar{\nu }}^{t}R{\left|{\vec{\mu }}_{1}^{t}\right|}^{2}\cos\left(\theta \right)\sin\left(\theta \right)\sin\left(\omega \right)$$

In a similar way, for the E1 and E2 states, we obtain Formula (57), which turns into (58) when the symmetry restrictions for the *R*_j_ and *μ*_j_ vectors are applied.

(57) RE1=πν¯t3Im{i[e−i2π/3(R→2−R→1)(μ→1×μ→2)+ei2π/3(R→3−R→1)(μ→1×μ→3)+ei4π/3(R→3−R→2)(μ→2×μ→3)]}RE2=πν¯t3Im{i[ei2π/3(R→2−R→1)(μ→1×μ→2)+e−i2π/3(R→3−R→1)(μ→1×μ→3)+e−i4π/3(R→3−R→2)(μ→2×μ→3)]}

(58) $$R^{E1}=R^{E2}=\frac{3\pi {\bar{\nu }}^{t}R{\left|{\vec{\mu }}_{1}^{t}\right|}^{2}}{2}\cos\left(\theta \right)\sin\left(\theta \right)\sin\left(\omega \right)$$

We notice that, in line with the results already known for binary systems, the transition to a totally-symmetric state (A in this case) and those to less symmetric states display opposite rotatory strengths. Furthermore, when the two possible E transitions are considered, the absolute values of this quantity are equivalent. The same trend is observed in the remaining symmetries herein considered.

#### 2.2.2. *D*_3_ Geometries

In a *D*_3_ arrangement, the totally-symmetric species is represented as A_1_ (ground electronic state). There are again three equivalent monoexcitations given by (37). They form a suitable basis set for constructing *D*_3_ SALCs for the first electronically-excited states. The reducible representation of this basis, $\Gamma^{a}$, decomposes into different symmetry-irreducible species depending on how the rotation around any of the three perpendicular *C*_2_ axes affects the first electronically-excited state of the isolated chromophore, $\psi^{a}$. When ${\hat{C}}_{2}\psi^{a}=\psi^{a}$, the typical situation when all the MOs describing the excited state are σ $\Gamma^{a}$ decomposes into A_1_ and the truly degenerate reducible species E. In contrast, when the excited state of the chromophore contains one π MO: ${\hat{C}}_{2}\psi^{a}=-\psi^{a}$, and $\Gamma^{a}$ contains A_2_ and E species. Thus, the two degenerate E excited electronic states, represented in the zero-order approach by any linear combination of Equations (61) and (62), can be accompanied by one A_1_ or A_2_ state, represented by a common expression, equivalent to that shown above for *C*_3_ systems (39). 

The transition from the ground state is orbitally allowed for the three states only when the excited state considered for the isolated chromophore is antisymmetric for *C*_2_ rotations (e.g., π* states). In this case, the application of the first-order perturbation theory leads to the same splitting of the excited level obtained for *C*_3_ geometries: $3V_{12}^{tt}$. On the other hand, when *C*_2_ rotations are symmetric (this is the case of σ* states), the transition to the A_1_ excited state is orbitally forbidden, and there is no splitting.

(59) $$\Psi_{E1}^{a\left(0\right)}=\frac{1}{2}\left[2\psi^{a}\psi^{0}\psi^{0}-\psi^{0}\psi^{a}\psi^{0}-\psi^{0}\psi^{0}\psi^{a}\right]$$

(60) $$\Psi_{E2}^{a\left(0\right)}=\frac{1}{\sqrt{2}}\left[\psi^{0}\psi^{a}\psi^{0}-\psi^{0}\psi^{0}\psi^{a}\right]$$

The presence of three *C*_2_ axes orthogonal to *C*_3_ restrains the orientations for EDTMs in *D*_3_ systems with regard to *C*_3_ ones to those where *ω* = π/2. As a consequence, the EDTM for the transition to the A_2_ state follows the same expression obtained for *C*_3_ symmetry (49), standing along the main symmetry axis, whereas the EDTMs for transitions to true degenerate E states are contained within the orthogonal plane, being linear combinations of the vectors represented by (61) and (62).

(61) $${\vec{\mu }}^{tE1}=\frac{3\left|{\vec{\mu }}_{1}^{t}\right|\sin\left(\theta \right)}{2}\vec{i}$$

(62) $${\vec{\mu }}^{tE2}=\sqrt{\frac{3}{2}}\left|{\vec{\mu }}_{1}^{t}\right|\sin\left(\theta \right)\vec{j}$$

Furthermore, the MDTM involved in the transition to the A_2_ state follows the same expression as that to the A excited state in *C*_3_ symmetry (52). Consequently, the expression for the rotatory strength for this transition is common for *C*_3_ and *D*_3_ symmetries (56). Transitions to E states display, in contrast, different expressions for MDTMs in *D*_3_ systems (linear combinations of (63) and (64)) and rotatory strength ((65) and (66)).

(63) $${\vec{M}}^{tE1}=\frac{i\pi {\bar{\nu }}^{t}}{2}\left(2{\vec{R}}_{1}\times {\vec{\mu }}_{1}^{t}-{\vec{R}}_{2}\times {\vec{\mu }}_{2}^{t}-{\vec{R}}_{3}\times {\vec{\mu }}_{3}^{t}\right)$$

(64) $${\vec{M}}^{tE2}=\frac{i\pi {\bar{\nu }}^{t}}{\sqrt{2}}\left({\vec{R}}_{2}\times {\vec{\mu }}_{2}^{t}-{\vec{R}}_{3}\times {\vec{\mu }}_{3}^{t}\right)$$

(65) $$R^{E1}=\frac{\pi {\bar{\nu }}^{t}}{4}\left\{2\left[\left({\vec{R}}_{2}-{\vec{R}}_{1}\right)\left({\vec{\mu }}_{1}\times {\vec{\mu }}_{2}\right)+\left({\vec{R}}_{3}-{\vec{R}}_{1}\right)\left({\vec{\mu }}_{1}\times {\vec{\mu }}_{3}\right)\right]-\left({\vec{R}}_{3}-{\vec{R}}_{2}\right)\left({\vec{\mu }}_{2}\times {\vec{\mu }}_{3}\right)\right\}=\frac{3\pi {\bar{\nu }}^{t}{\left|{\vec{\mu }}_{1}^{t}\right|}^{2}R}{2}\sin\left(\theta \right)\cos\left(\theta \right)$$

(66) $$R^{E2}=\frac{\pi {\bar{\nu }}^{t}}{2}\left({\vec{R}}_{3}-{\vec{R}}_{2}\right)\left({\vec{\mu }}_{2}\times {\vec{\mu }}_{3}\right)=\frac{3\pi {\bar{\nu }}^{t}{\left|{\vec{\mu }}_{1}^{t}\right|}^{2}R}{2}\sin\left(\theta \right)\cos\left(\theta \right)$$

#### 2.2.3. *C*_4_ and *D*_4_ Geometries

**Scheme 6:**
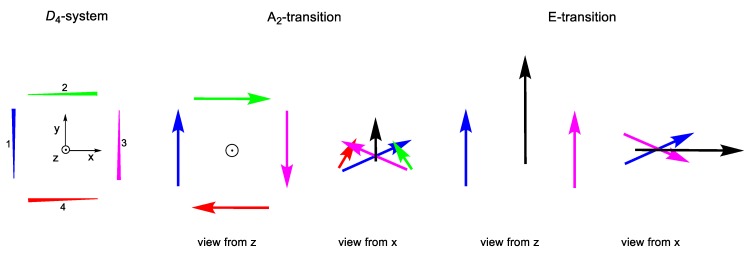
Representation of a system presenting *D*_4_ symmetry. According to the selection rules, the four equivalent chromophores 1 (blue), 2 (magenta), 3 (green), and 4 (red) can undergo a simultaneous excitation following A_2_ or E symmetry leading to total EDTMs (black) parallel to z or y, respectively, as a result of the summation of the EDTM of each chromophore. A circle with a dot in the middle represents an arrow perpendicular to the plane pointing to the reader.

Considering a *C*_4_ structure including four chromophores (Scheme 6), a pairwise approach for the interaction among them, where V_12_ and V_13_ are not equivalent, (27) the same approximations, and a similar treatment to that shown above (see Supporting Information) leads to the following conclusions: (i) there are four monoexcited states, two of them pseudodegenerate, belonging to the E symmetry species, the other with A and B symmetry; (ii) the electronic transition from the ground state to the B one is orbitally forbidden, whereas those to A and E are allowed; and (iii) the energy of state A differs from those of E (equivalent within the first-order approach) in $2\left(V_{12}^{tt}+V_{13}^{tt}\right)$; which is the splitting in this case.

(67) $$\hat{V}=4{\hat{V}}_{12}+2{\hat{V}}_{13}$$

As in the three-chromophore case, replacing *C*_n_ symmetry by *D*_n_ splits some symmetry species (A into A_1_ and A_2_ and B into B_1_ and B_2_), while pseudodegenerate E states become degenerate. A_1_ and B_1_ species are symmetric for *C*_2_ rotations over any perpendicular axis and correspond to excited states with forbidden transitions from the ground state. In contrast, A_2_ and B_2_ states are antisymmetric for these perpendicular rotations, and the transitions between the ground state and A_2_ are orbitally-allowed. As a consequence, the excited state splitting is only observed for excited states involving the excitation of one chromophore to an antisymmetric state with regard to a perpendicular rotation. In this case, the energy of state A_2_ differs from the E ones again by $2\left(V_{12}^{tt}+V_{13}^{tt}\right)$ (see the Supporting Information).

Similarly, the expressions for molecular EDTM in *C*_4_ systems ((68) and (69)) show that the absorption for 0→A and 0→E transitions depends on the molecular geometry. This can be described in terms of *θ* and *ω* angles, defined as in C_3_ systems, but now identifying the z axis with C_4_ and considering the recursive formula *ω_j+1_ = ω_j_ + π/2* for the series of *ω* angles of the chromophores. 

MTDMs for the transitions E and A in a *C*_4_ system follow, respectively, Equations (70) and (71). Thus, we arrive at Expressions (72) and (73) for the corresponding rotatory strength.

(68) $${\vec{\mu }}^{tA}=2\left|{\vec{\mu }}_{1}^{t}\right|\cos\left(\theta \right)\vec{k}$$

(69) $$\begin{array}{c} {\vec{\mu }}^{tE1}=\;\left|{\vec{\mu }}_{1}^{t}\right|\sin\theta \left[\left(\cos\omega -i\sin\omega \right)\;\vec{i}+\left(\sin\omega +i\cos\omega \right)\;\vec{j}\right]\\ {\vec{\mu }}^{tE2}=\;\left|{\vec{\mu }}_{1}^{t}\right|\sin\theta \left[\left(\cos\omega +i\sin\omega \right)\;\vec{i}+\left(\sin\omega -i\cos\omega \right)\;\vec{j}\right] \end{array}$$

(70) $$\begin{array}{c} {\vec{M}}^{tE1}=\frac{\pi {\bar{\nu }}^{t}}{2}\left[i\left({\vec{R}}_{1}\times {\vec{\mu }}_{1}^{t}-{\vec{R}}_{3}\times {\vec{\mu }}_{3}^{t}\right)-\left({\vec{R}}_{2}\times {\vec{\mu }}_{2}^{t}-{\vec{R}}_{4}\times {\vec{\mu }}_{4}^{t}\right)\right]\\ {\vec{M}}^{tE2}=\frac{\pi {\bar{\nu }}^{t}}{2}\left[i\left({\vec{R}}_{1}\times {\vec{\mu }}_{1}^{t}-{\vec{R}}_{3}\times {\vec{\mu }}_{3}^{t}\right)+\left({\vec{R}}_{2}\times {\vec{\mu }}_{2}^{t}-{\vec{R}}_{4}\times {\vec{\mu }}_{4}^{t}\right)\right] \end{array}$$

(71) $${\vec{M}}^{tA}=\frac{i\pi {\bar{\nu }}^{t}}{2}\left({\vec{R}}_{1}\times {\vec{\mu }}_{1}^{t}+{\vec{R}}_{2}\times {\vec{\mu }}_{2}^{t}+{\vec{R}}_{3}\times {\vec{\mu }}_{3}^{t}+{\vec{R}}_{4}\times {\vec{\mu }}_{4}^{t}\right)$$

(72) $$R^{E1}=R^{E2}=2\pi {\bar{\nu }}^{t}{\left|{\vec{\mu }}_{1}^{t}\right|}^{2}R\sin\theta \cos\theta \sin\omega$$

(73) $$R^{A}=\frac{-\pi {\bar{\nu }}^{t}}{4}\sum_{j=1}^{3}\sum_{k=j+1}^{4}\left({\vec{R}}_{k}-{\vec{R}}_{j}\right)\left({\vec{\mu }}_{j}^{t}\times {\vec{\mu }}_{k}^{t}\right)=-4\pi {\bar{\nu }}^{t}{\left|{\vec{\mu }}_{1}^{t}\right|}^{2}R\sin\theta \cos\theta \sin\omega$$

Imposing *D*_4_ symmetry (*ω* = π/2 for Chromophore 1) does not alter any of the expressions shown for transition A. Nevertheless, it allows obtaining vectors with real components for EDTMs and imaginary ones for MDTMs to E degenerate states. These vectors are linear combinations of those shown respectively in (74) and (75). The former is always contained in the orthogonal plane to the *C*_4_ axis, whereas the latter are parallel to it.

(74) $${\vec{\mu }}^{tE1}=\frac{{\vec{\mu }}_{1}^{t}-{\vec{\mu }}_{3}^{t}}{\sqrt{2}}=\sqrt{2}\sin\left(\theta \right)\vec{i};\;{\vec{\mu }}^{tE2}=\frac{{\vec{\mu }}_{2}^{t}-{\vec{\mu }}_{4}^{t}}{\sqrt{2}}=\sqrt{2}\sin\left(\theta \right)\vec{j}$$

(75) $$\begin{array}{l} {\vec{M}}^{tE1}=\frac{i\pi {\bar{\nu }}^{t}}{\sqrt{2}}\left({\vec{R}}_{1}\times {\vec{\mu }}_{1}^{t}-{\vec{R}}_{3}\times {\vec{\mu }}_{3}^{t}\right);\\ {\vec{M}}^{tE2}=\frac{i\pi {\bar{\nu }}^{t}}{\sqrt{2}}\left({\vec{R}}_{2}\times {\vec{\mu }}_{2}^{t}-{\vec{R}}_{4}\times {\vec{\mu }}_{4}^{t}\right) \end{array}$$

Finally, the expressions for rotatory strength can be simplified taking into account the ω restriction.

(76) $$R^{E}=\frac{\pi {\bar{\nu }}^{t}}{2}\left({\vec{R}}_{3}-{\vec{R}}_{1}\right)\left({\vec{\mu }}_{1}^{t}\times {\vec{\mu }}_{3}^{t}\right)=2\pi {\bar{\nu }}^{t}{\left|{\vec{\mu }}_{1}^{t}\right|}^{2}R\sin\theta \cos\theta$$

(77) $$R^{A}=\frac{-\pi {\bar{\nu }}^{t}}{4}\sum_{j=1}^{3}\sum_{k=j+1}^{4}\left({\vec{R}}_{k}-{\vec{R}}_{j}\right)\left({\vec{\mu }}_{j}^{t}\times {\vec{\mu }}_{k}^{t}\right)=-4\pi {\bar{\nu }}^{t}{\left|{\vec{\mu }}_{1}^{t}\right|}^{2}R\sin\theta \cos\theta$$

### 2.3. Comparison with DFT Calculations

**Scheme 7:**
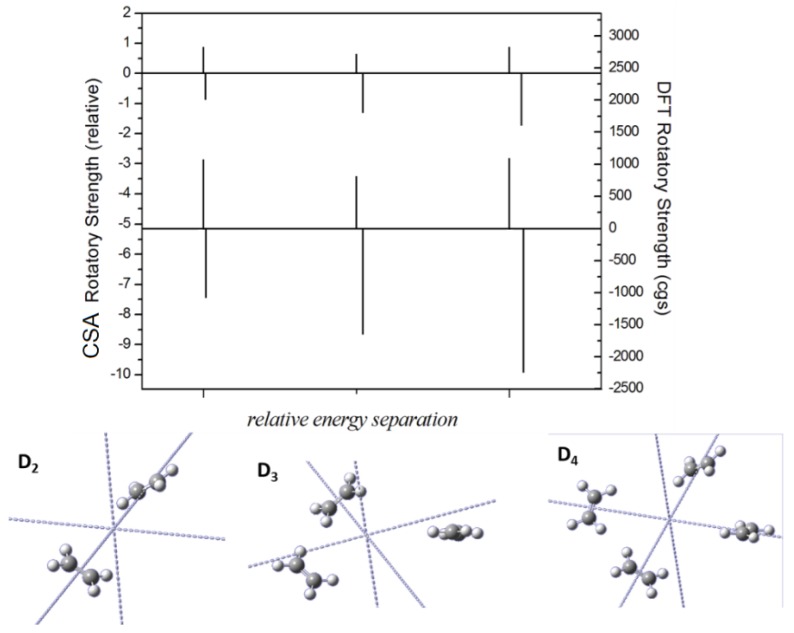
Top: DFT predicted (graph bottom) and CSA predicted (graph top) rotatory strengths for *D*_2_, *D*_3_, and *D*_4_ (from left to right) symmetries. The bottom axis represents the relative energy for each symmetry (from left to right: higher to lower). Bottom: *D*_n_ systems.

The rotatory strength for the two lower energetic transitions were calculated with DFT and CSA for three systems bearing two, three, and four ethene moieties with a 5 Å distance to the center of mass and a 30° torsion angle with respect to the y axis with *D*_2_, *D*_3_, and *D*_4_ symmetry, respectively (Scheme 7).

The sign of the energy difference between the two allowed transitions, which is reflected on the order of the two rotatory strengths at the spectrum, was correctly predicted by the CSA model giving the same chirality as the DFT calculations. Even when the values calculated from the CSA were only relative, they led to the same relative intensities as compared to the DFT calculations.

## 3. Conclusions

It has been shown how the Chiroptical Symmetry Analysis (CSA) may significantly contribute to uncovering the chiroptical responses of highly-symmetric systems bearing independent chromophores given that: (i) V_ij_ can be calculated according to the Davydov equation; (ii) the allowed transitions can be predicted by applying the selection rules; (iii) Δ*E* between those transitions can be calculated according to theory; (iv) the symmetry of the allowed transitions enables simple calculation of the total EDTM, MDTM, and rotatory strength associated.

This approach is not intended to predict the experimental CD response of a particular system accurately, but rather to be employed as a useful tool for the design of valuable systems for chiroptical applications. Next, we intend to develop software for a straightforward analysis of diverse systems to explore the scope of the Chiroptical Symmetry Analysis (CSA).

## Supplementary Materials

The following are available online: S0. Obtaining molecular EDTMs from those of isolated chromophores in a binary system. S1. Excited state splitting for C4 geometries. S2. Excited state splitting for D4 geometries. S3. Deriving the relationship between the ${\vec{p}}^{t}$ and ${\vec{\mu }}^{t}$ vectors.
