# Supplementary material for: Chiroptical Symmetry Analysis: Exciton Chirality-Based Formulae to Understand the Chiroptical Responses of Cn and Dn Symmetric Systems
Source: Molecules. 2019 Jan 1;24(1):141. doi: 10.3390/molecules24010141 (PMC6337451; doi:10.3390/molecules24010141)
Supplement: Supplementary file 1 [file molecules-24-00141-s001.pdf]

molecules

S0. Obtaining molecular EDTMs from those of isolated chromophores in a binary system.

Molecular EDTM for the transition from ground, Ψ^0, to a certain electronic excited state with Ψ^a wavefunction is defined by equation (S0.1). Assuming that, in a binary system made up of two chromophores, wavefunctions of both ground electronic and excited electronic state (α or β) (a 9 can be approached by zero-order wavefunctions, as given by equations (4), (12), and (13), gives rise to expressions (S0.2) and (S0.3) for EDTMs to α and β states. The electric dipole moment operator can be split into two parts, the first affecting exclusively to the electrons contained in chromophore 1, and the second to those of chromophore 2, as shown in (S0.4). This leads to equations (S0.5) and (S0.6).

$$\vec{\mu}^{ta} = \langle \Psi^0 | \hat{\mu}_e | \Psi^a \rangle \tag{S0.1}$$

$$\vec{\mu}^{t\alpha} = \frac{1}{\sqrt{2}} \langle \psi^0 \psi^0 | \hat{\mu}_e | (\psi^a \psi^0 - \psi^0 \psi^a) \rangle \tag{S0.2}$$

$$\vec{\mu}^{t\beta} = \frac{1}{\sqrt{2}} \langle \psi^0 \psi^0 | \hat{\mu}_e | (\psi^a \psi^0 + \psi^0 \psi^a) \rangle \tag{S0.3}$$

$$\hat{\mu}_e = e \sum_{j=1}^{2} \sum_{k=1}^{K} \vec{r}_{jk} = e \sum_{k=1}^{K} \vec{r}_{1k} + e \sum_{k=1}^{K} \vec{r}_{2k} = \hat{\mu}_1 + \hat{\mu}_2 \tag{S0.4}$$

$$\vec{\mu}^{t\alpha} = \frac{1}{\sqrt{2}} \left[\langle \psi^0 | \hat{\mu}_1 | \psi^a \rangle \langle \psi^0 | \psi^0 \rangle - \langle \psi^0 | \psi^0 \rangle \langle \psi^0 | \hat{\mu}_2 | \psi^a \rangle \right], \tag{S0.5}$$

$$\vec{\mu}^{t\beta} = \frac{1}{\sqrt{2}} \left[\langle \psi^0 | \hat{\mu}_1 | \psi^a \rangle \langle \psi^0 | \psi^0 \rangle + \langle \psi^0 | \psi^0 \rangle \langle \psi^0 | \hat{\mu}_2 | \psi^a \rangle \right] \tag{S0.6}$$

Finally, introducing EDTM for each chrompohore *j* (S0.7), (S0.5) and (S0.6) turn into expressions (20) and (21).

$$\vec{\mu}_j^t = \langle \psi^0 | \hat{\mu}_j | \psi^a \rangle, \tag{S0.7}$$

S1. Excited state splitting for C_4 geometries.

The wave function for the electronic ground state is given, within the zero-order approximation by equation (S1.1) and belongs to the totally symmetric irreducible representation A. The functions shown in (S1.2) allow expanding the four independent C_4 SALCs for the first excited level in this approach: two of them are pseudodegenerate and belong to the E irreducible symmetry species, whereas the other are of A and B symmetry (equations S1.3 to S1.6, respectively). The transitions from the totally symmetric ground state are only orbitally-allowed to excited states which have the same symmetry of, at least, one of the components of the dipole moment operator. Therefore, the transition to the B state is forbidden, whereas those to A, E1, and E2 are allowed.

$$\Psi^{0(0)} = \psi^0\psi^0\psi^0\psi^0 \tag{S1.1}$$

$$\begin{gathered}\Psi_1^{a(0)} = \psi^a\psi^0\psi^0\psi^0, \quad \Psi_2^{a(0)} = \psi^0\psi^a\psi^0\psi^0, \\ \Psi_3^{a(0)} = \psi^0\psi^0\psi^a\psi^0, \Psi_4^{a(0)} = \psi^0\psi^0\psi^0\psi^a, \end{gathered} \tag{S1.2}$$

$$\Psi_{E1}^{a(0)} = \frac{1}{2}\left[\left(\psi^a\psi^0\psi^0\psi^0 - \psi^0\psi^0\psi^a\psi^0\right) + i\left(\psi^0\psi^a\psi^0\psi^0 - \psi^0\psi^0\psi^0\psi^a\right)\right] \tag{S1.3}$$

$$\Psi_{E2}^{a(0)} = \frac{1}{2}\left[\left(\psi^a\psi^0\psi^0\psi^0 - \psi^0\psi^0\psi^a\psi^0\right) - i\left(\psi^0\psi^a\psi^0\psi^0 - \psi^0\psi^0\psi^0\psi^a\right)\right] \tag{S1.4}$$

$$\Psi_{A}^{a(0)} = \frac{1}{2}\left[\psi^a\psi^0\psi^0\psi^0 + \psi^0\psi^a\psi^0\psi^0 + \psi^0\psi^0\psi^a\psi^0 + \psi^0\psi^0\psi^0\psi^a\right] \tag{S1.5}$$

$$\Psi_{B}^{a(0)} = \frac{1}{2}\left[\psi^a\psi^0\psi^0\psi^0 - \psi^0\psi^a\psi^0\psi^0 + \psi^0\psi^0\psi^a\psi^0 - \psi^0\psi^0\psi^0\psi^a\right] \tag{S1.6}$$

Application of the first order perturbation theory, when the potential energy is given by (27), gives rise to identical corrections for the energy of E1 and E2 states, given by (S1.7), whereas that of the A excited state is represented by (S1.8).

$$E_{E1}^{a(1)} = E_{E2}^{a(1)} = 2\left(V_{12}^{a0} + V_{12}^{00}\right) + \left(V_{13}^{a0} + V_{13}^{00} - V_{13}^{tt}\right) \tag{S1.7}$$

$$E_{A}^{a(1)} = 2\left(V_{12}^{a0} + V_{12}^{00} + V_{12}^{tt}\right) + \left(V_{13}^{a0} + V_{13}^{00} + V_{13}^{tt}\right) \tag{S1.8}$$

S2. Excited state splitting for D_4 geometries.

The wave function for the electronic ground state displays, within the zero-order approximation, the same expression shown above for C_4 symmetry (S1.1), and belongs to the totally symmetric irreducible representation A_1. The functions shown in (S1.2) allow expanding the four independent D_4 SALCs for the first excited level in this approach. In this case, as it happens with D_3-symmetry, it is necessary two distinguish two possibilities: i) chromophores symmetric for C_2' rotations; and ii) chromophores antisymmetric for C_2' rotations, which can be, respectively exemplified by σ and π chromophores. The reducible representation for case i) contains A_1, B_1 and E representations, whereas for case ii) contains A_2, B_2 and E. As a consequence, D_4 symmetry structures with σ chromophores display a single orbitally-allowed electronic transition to the first set of excited states. In contrast, when the chromophores are π the single transition is replaced by two bands, representing the transitions from the A_1 ground level to A_2 and E states, whose wave functions are, represented by equation (S2.1) (A_1) and any linear combination of (S2.2) and (S2.3) (E).

$$\Psi_{A_2}^{a(0)} = \frac{1}{2}\left[\psi^a\psi^0\psi^0\psi^0 + \psi^0\psi^a\psi^0\psi^0 + \psi^0\psi^0\psi^a\psi^0 + \psi^0\psi^0\psi^0\psi^a\right] \tag{S2.1}$$

$$\Psi_{E1}^{a(0)} = \frac{1}{\sqrt{2}}\left(\psi^a\psi^0\psi^0\psi^0 - \psi^0\psi^0\psi^a\psi^0\right) \tag{S2.2}$$

$$\Psi_{E2}^{a(0)} = \frac{1}{\sqrt{2}}\left(\psi^0\psi^a\psi^0\psi^0 - \psi^0\psi^0\psi^0\psi^a\right) \tag{S2.3}$$

Application of the first order perturbation theory, when the potential energy is given by (27), gives rise to a correction for the energy of E states, given by (S2.4), whereas that of the A_1 is represented by (S2.5).

$$E_E^{a(1)} = 2\left(V_{12}^{a0} + V_{12}^{00}\right) + \left(V_{13}^{a0} + V_{13}^{00} - V_{13}^{tt}\right) \tag{S2.4}$$

$$E_{A_2}^{a(1)} = 2\left(V_{12}^{a0} + V_{12}^{00} + V_{12}^{tt}\right) + \left(V_{13}^{a0} + V_{13}^{00} + V_{13}^{tt}\right) \tag{S2.5}$$

S3. Deriving the relationship between $\vec{p}^{\,t}$ and $\vec{\mu}^{\,t}$ vectors.

Simple relations of operators algebra outlined below (equations (S3.1) to (S3.3)) allow to replace $\vec{p}_j^{\,t}$ by the EDTM of the isolated chromophore, $\vec{\mu}_j^{\,t}$, through expressions involving the wave number for the transition, ν^t.

$$\begin{aligned}\left\langle\psi^0\left|\left[\hat{H},\vec{r}_k\right]\right|\psi^a\right\rangle &= \left\langle\psi^0\left|\hat{H}\right|\vec{r}_k\psi^a\right\rangle - \left\langle\psi^0\left|\vec{r}_k\right|\hat{H}\psi^a\right\rangle = \\ &= \left\langle\vec{r}_k\psi^a\left|\hat{H}\right|\psi^0\right\rangle - \left\langle\psi^0\left|\vec{r}_k\right|\hat{H}\psi^a\right\rangle = \left(E^0 - E^a\right)\left\langle\psi^0\left|\vec{r}_k\right|\psi^a\right\rangle = \frac{\left(E^0 - E^a\right)\vec{\mu}^t}{e}\end{aligned} \tag{S3.1}$$

$$\begin{aligned}\left\langle\psi^0\left|\left[\hat{H},\vec{r}_k\right]\right|\psi^a\right\rangle &= \left\langle\psi^0\left|\left[\left(\frac{\hat{p}_k^2}{2m}+\hat{V}\right),\vec{r}_k\right]\right|\psi^a\right\rangle = \frac{1}{2m}\left\langle\psi^0\left|\left[\hat{p}_k\hat{p}_k,\vec{r}_k\right]\right|\psi^a\right\rangle = \\ &= \frac{1}{2m}\left\langle\psi^0\left|\hat{p}_k\hat{p}_k\vec{r}_k - \vec{r}_k\hat{p}_k\hat{p}_k + \hat{p}_k\vec{r}_k\hat{p}_k - \hat{p}_k\vec{r}_k\hat{p}_k\right|\psi^a\right\rangle = \frac{1}{2m}\left\langle\psi^0\left|\hat{p}_k\left[\hat{p}_k,\vec{r}_k\right] - \left[\vec{r}_k,\hat{p}_k\right]\hat{p}_k\right|\psi^a\right\rangle = \frac{\hbar}{mi}\vec{p}^{\,t}\end{aligned} \tag{S3.2}$$

$$\vec{p}^{\,t} = \frac{mi\left(E^0 - E^a\right)}{e\hbar}\vec{\mu}^t = \frac{2\pi mci\,\nu^t}{e}\vec{\mu}^t \tag{S3.3}$$
